# A Cohort-Based Case Report: The Impact of Ketamine-Assisted Therapy Embedded in a Community of Practice Framework for Healthcare Providers With PTSD and Depression

**DOI:** 10.3389/fpsyt.2021.803279

**Published:** 2022-01-12

**Authors:** Shannon Dames, Pamela Kryskow, Crosbie Watler

**Affiliations:** ^1^Health and Human Services, Vancouver Island University, Nanaimo, BC, Canada; ^2^Private Practice, Duncan, BC, Canada

**Keywords:** ketamine-assisted psychotherapy, healthcare providers (HCP), resilience, psychedelic therapy, group therapy, community of practice, post-traumatic stress disorder (PTSD), depression

## Abstract

Amid an international pandemic and a worsening mental health crisis, ketamine-assisted therapy is emerging as a promising solution for those deemed “treatment resistant.” Post-traumatic stress disorder (PTSD) and depression are on the rise, with accelerating direct (e.g., burden of suffering) and indirect (e.g., disability/role impairment and impact on family) costs. Psychedelic-assisted therapies show significant promise in the treatment of a number of clinically challenging conditions, including depression, anxiety, PTSD, addiction, and end-of-life distress. Ketamine is currently the only safe, effective and legal widely available psychedelic-like medicine. To address the echo pandemic of health care provider distress, a multi-disciplinary team was charged with developing a ketamine-assisted psychotherapy program, delivered in a community of practice (CoP) group model and evaluated in a quality improvement framework. Program evaluation occurred through mixed methods. Quantitative mental health assessments included the PHQ-9 for depression, the PCL-5 for PTSD, GAD-7 for generalized anxiety disorder (GAD), and B-IPF for work/life functionality. Participant narrative feedback was collected to evaluate outcomes and for quality improvement purposes. Mean mental health scores were collected across three cohorts, totaling 94 patients. The mean aggregate scores of participants meeting the mental health assessment cut-off criteria (screening positive) were analyzed to assess clinical significance. Mean aggregate results comparing baseline vs. outcome measures (measured within 1–2 weeks after completion of the 12-week program) were clinically significant, demonstrating significant improvements in depression, post-traumatic stress disorder, generalized anxiety disorder and work/life functionality. In summary, 91% saw improvements in generalized anxiety, 79% saw improvements in depression, 86% of those who screened positive for PTSD now screen negative, and 92% had significant life/work functionality improvements. Qualitative feedback was overwhelmingly positive, with several unsolicited self-reports of transformation. Participant and team feedback enables the program to continue improving with each iteration. Results speak to the effectiveness of ketamine for psychedelic-assisted therapy, supported by a CoP framework. Outcomes are relevant for mental health programming, education and healthcare policy.

## Introduction

The impetus for embedding ketamine-assisted therapy (KaT) within a group-oriented community of practice (CoP) revolves around the growing mental health crises in Canada, with healthcare providers (HCP) disproportionately impacted (1). Due to high stimulus and trauma-laden work environments, they are at a higher risk for mental health challenges (2). Even before the pandemic, 40–60% of HCPs were experiencing burnout at some point in their career (3), and the addition of COVID-19 is causing a devastating impact on morale, absenteeism, retention and patient care (1). Given HCP training and lived experience, their input helps identify occupational health and safety gaps and options for remediation.

## A Call for Innovation

Modern psychiatry was strongly influenced by the emergence of so-called *Biological Psychiatry* and the *Decade of the Brain (1990's)*. The belief at the time was that we were on the threshold of a new psychiatry, where we could reduce mental illness to defined perturbations of neuroreceptors, trusting that our novel *anti*depressants and *anti*psychotics would provide definitive treatment.

By any standard, this has been a failed experiment. Despite an exponential increase in prescribing over the recent decades, even before the pandemic, the costs of mental health disability were higher than ever before and steadily rising (4), with depression as the leading cause (5). Treatment wise, it is common knowledge that the placebo response to antidepressants is equal to, or greater than the effect of the medication (6). Further, among those who respond, many are left with subsyndromal symptoms, with only a minority realizing full remission. Not surprisingly, there is a growing cohort of *treatment resistant* patients. Perhaps our patients are not treatment resistant at all, perhaps we are simply using the wrong treatments.

Modern psychiatry is failing to get to the source of the *distress* that is a product of living in an increasingly frenetic and disconnected world. Instead, we label distress as *disorder*, failing to recognize that many of our disorders are simply an adaptation to increasingly hostile and toxic environments. The failure of frontline therapies for a growing number of people is accelerating the re-emergence of psychedelic-assisted therapies.

### The Re-emergence Psychedelic Agents

Psychedelic-assisted therapies are demonstrating positive outcomes in the treatment of depression, PTSD, addiction, and end-of-life distress (7). They are radically different from traditional therapy, requiring specific training to support the client preparing for, navigating and integrating transformation from non-ordinary states of consciousness. Unique to psychedelic therapy, is the interruption of autonomic nervous system's fight-flight-freeze reactions, allowing recipients to re-orient themselves to traumatic events and disempowering belief systems of the past returning to a parasympathetic state.

To safely incorporate these new therapies, the current biomedical model of Canada's public healthcare system will need significant capacity-building. Currently, ketamine is the only legal and widely available psychedelic-like medicine. Ketamine is an FDA-approved drug, mainly used as an anesthetic in high doses with persons with MDD and PTSD (8). When ketamine is administered in sub-anesthetic doses, it produces a phenomenological psychedelic effect similar in some ways to psilocybin and Lysergic acid diethylamide (LSD) (9), enabling therapists to help people work with and through the belief systems and emotions associated with trauma. KaT shows significantly positive outcomes treating PTSD, depression, and addictions (10–12). As a result, working with ketamine provides an opportunity to provide psychedelic-assisted therapy, enabling a translation of knowledge to other psychedelic medicines as they become available. Finally, developing and delivering psychedelic-assisted therapy programs in multidisciplinary holistic teams will maximize therapeutic impact and minimize safety risks.

## Developing the Innovative Treatment Plan

An interdisciplinary and multi-agency team innovated by combining KaT, using ketamine as a psychedelic-like agent, with an evidence-informed CoP program, serves HCPs facing depression and PTSD. The program was a registered quality improvement project. The community based, evidence and equity informed framework is designed to minimize safety risks and to support and maintain recovery. The team's guiding principles include holistic, trauma-informed care, inclusivity, cultural/personal safety and constant quality improvement. Aligned with Canada's Strategy for Patient-Oriented Research led by the Canadian Institutes of Health Research our program is about engaging HCPs in the research process. This engagement helps to ensure that studies focus on HCP-identified priorities, which ultimately lead to better treatments and patient outcomes. The treatment program is 12-weeks in length (2 h each week, three, 4-h sessions for KaT; ~40 h in total) and runs through Vancouver Island University.

## Building the Interdisciplinary Team

The team includes medical doctors, researchers, HCPs with lived experience of depression and PTSD, nurses, clinical counselors, spiritual health practitioners, Indigenous Elders and Knowledge Holders, Somatic Energy Practitioners, Functional Medicine Experts, Sleep Experts, Aromatherapy experts, and Physical Movement Experts. This style of team is rare in the Western Medical Model, necessitating apprenticeship/mentoring to build the team and orient new members.

Alongside Indigenous knowledge holders and cultural safety experts, the team engages in ongoing training and practices that promote equity and trauma informed care. The team models the program's guiding principles, including the adoption of shared intentions and agreements. These include, honesty, authenticity, vulnerability, and the mirroring of unconditional positive regard. The realization of these practices and principles bring forth a remarkable real humanity and palpable caring within the team. This, in turn, creates an environment of safety and trust that is often espoused, but rarely realized in mainstream health care settings.

Those wishing to join the team are encouraged to first go through the program as participants in the CoP program, and if eligible, in the KaT portion as well. Once participants transition to alumni, they can apply to be a mentee on the team, working closely with a mentor before they become a full facilitating team member.

## Intake and Screening Process

HCPs with depression and PTSD were the priority population. Prior to acceptance into the program, participants first undergo a thorough three step intake process. The initial intake is completed by a registered psychiatric nurse who introduces participants to the program concepts through regulation practices, inner attunement, and authentic expression. For instance, the intake begins with a breathing technique that promotes relaxation, encouraging them to listen to what is arising in their physical awareness, and to speak to their fears and hopes of the process.

If determined a good fit by the participant-candidate and the team member, they proceed to meet with the team psychiatrist. If cleared by the team's psychiatrist, they then proceed to a meeting with a medical doctor who specializes in psychedelic-assisted therapy, ensuring no contraindications to ketamine use.

### Inclusion Criteria

Referral from a primary care provider (or provider with a longitudinal relationship with client) *AND* history of previous treatment failure *AND* diagnosis of treatment resistant depression (11, 13), chronic anxiety (14), obsessive-compulsive disorder (OCD) (15), suicidality (16), PTSD (11), or Substance Use Disorder (10, 17).

### Exclusion Criteria

Hypersensitivity to ketamine, presence of active psychotic symptoms, diagnosis of dementia/delirium, high risk for coronary artery disease, uncontrolled cardiopulmonary disease/cardiovascular disease/hypertension, aneurysm, history of intracerebral hemorrhage, hepatic cirrhosis, hepatorenal disease, recent changes in medications related to mood disorders, or pregnancy or if breastfeeding within 11 h of ketamine administration (18). Extreme emotional lability can be disruptive to the group milieu and is a relative exclusion criterion.

### Participant Feedback

“*The 3 intakes were very helpful in connecting with the various members of the team, and clarifying my intentions to participate. I appreciated the opportunity to ask questions, and have a one-to-one conversation with each intake team/facilitator.”*

## Treatment Mechanism: Community of Practice

The CoP curriculum is informed by the literature, describing what humans need to thrive and maintain resilience amidst adversity (2). The concept of a CoP was coined by Lave and Wenger (19) and defined by (20) as “groups of people who share a concern or a passion for something they do and learn how to do it better as they interact regularly (p. 1).” The program was developed from a research-informed resilience development framework (2) call *Roots to Thrive*, which focuses on the development of:

- congruence (21), representing one's orientation to self and authentic expression in the world, and
- sense of coherence (22), representing one's orientation to the world, bolstering meaning, understanding, and confidence in one's resources.

The context for developing these qualities favors a focus on the somatic nature of healing and present moment experience, within a CoP that mirrors unconditional positive regard (23) rather than sharing extended narratives and events of personal history. Aligning with Polyvagal Theory, when social relationships become a source of connection and security, they regulate the nervous system and encourage authentic ways of being (2, 24). The mechanisms of healing that are integrated into the CoP include:

- connection to self and others through the experience of secure attachment,
- addressing trauma within an environment of unconditional positive regard,
- regulating the nervous system,
- co-regulating through relationship,
- alignment with one's desires and calling.

Based on ongoing participant feedback, the most important healing components of the program revolve around the team's ability to model vulnerability themselves and to provide unconditional positive regard, which is quickly mirrored among participants.

### Participant Feedback

“*The small groups have been amazing. I look forward to weekly sessions. Getting to know these beautiful people in a different way has created a connection that I never knew could exist. How could we know each other without knowing all of our stories? The trust that was felt in our group occurred in the first session and has increased every week. I realized that I don't need to know their stories nor do they need to know mine to have a strong trusting relationship. I love that our facilitators are also sharing with us, they continue to work on themselves through this process.”*

### The Community of Practice Structure

The weekly CoP runs one evening a week for 2 h. The entire cohort (ranging from 50 to 100 participants) first meets in a large group format, then they move to the same breakout group each week for sharing. Each small group contains between six and nine participants and is facilitated by a clinician and therapist dyad. Except for the psychedelic medicine-assisted component of the 12-week program, all patient provider interactions are completed virtually. Additional sessions are provided by a Functional Medicine Practitioner, an Emotional Freedom Technique (EFT/Tapping) Practitioner, a Body Movement Practitioner (such as Yoga), and one to one support as needed from a variety of specialists on the team.

Each week, participants meet in a large group format for 30–40 min beginning with an opening message by the program's Indigenous Elder, followed by Program Logistics. A team member provides an evidence-informed *Coming To Know* teaching, which cultivates the understanding, meaning, confidence, and engagement necessary for the deeper work (at the level of the body) to unfold. All then engage in a Pause Practice (guided meditation or somatic self-regulation practice), which encourages transition from “figuring it out” at the level of the mind to a somatic or felt sense, honoring the inner healing intelligence within the body.

Within the breakout groups at the first meeting, each small group adopts a set of intentions and agreements. Members are accountable to one another, which is important for developing accountability, trust and a sense of relational security. Participants are paired with a *Buddy*, to connect and integrate with once a week, using a structured conversational format promoting authentic and embodied sharing.

During the small group break-out session, participants begin with a *Short Check-In*, where they name one to two physical sensations and emotions they feel in that moment. They then transition to a *Long Check-In*, where they speak to an open-ended reflective question that encourages contemplation, somatic reflection, and emotional expression. Participants receive an *Integration Practice* to incorporate over the following week, ending in a *Short Check-Out*, where they again name one to two physical sensations and emotions that they are experiencing in that moment.

All members are encouraged to engage according to their own comfort level, working within their unique window of tolerance for vulnerability. With practice, participants gain trust and confidence in the process and their small group. The mechanisms of healing in the CoP structure include co-regulation of the nervous system, somatic awareness, authentic expression, expanding one's window of tolerance for vulnerability and stress, and the giving and receiving of unconditional positive regard through compassionate witnessing (a verbal reflection practice from other group members to the person who shared, focusing on empathy and resonance).

### Participant Feedback

“*I am already experiencing moments of sadness when I think of these 12 sessions ending and I hope for it to continue in some form past the formal end of the program. I see that for integration to truly take place, I would need for the process to last longer than 12 weeks.”*

Upon completion of the program, participants receive an exit interview to reflect on the process and for continuity of care. For those wanting ongoing support after the 12-weeks, participants are invited to join an Alumni CoP Program, enabling ongoing peer and team support and additional access to KaT.

### Participant Feedback

“*I truly believe them when they tell me to ‘reach out anytime,' ‘we are always here for you' – the words, actions and energies all align leaving me with a deep sense of trust, and a willingness to open further in the safety of the container. I feel truly seen.”*

“*The delicate and thoughtful way in which the container of the group was developed was helpful and satisfying. I was amazed at how quickly I felt safe and willing to share my vulnerable pieces with the group. I have appreciated the offerings of various tools for my tool box.”*

## Treatment Mechanism: Three KaT Sessions

The KaT component of the 12-week program brings CoP members together to receive ketamine in a group format, with a skilled therapist-clinician dyad per six participants. In addition, floating cultural and spiritual safety experts and knowledge holders, somatic energy practitioners, medical doctors, and a psychiatrist oversee all of the group administrations, attending where needed.

KaT is typically provided at week 4, 5, and 7. The short window of time between sessions provides a liminal space that magnifies the psychedelic mechanism, which could also be described as a loosening of the mind/ego structures that lead to the sense of stuckness that characterizes many mental health conditions.

KaT sessions include at least one or more one-to-one preparatory sessions. Participants plan for the ketamine sessions to take 4 h. Participants arrive at the healthcare center and are welcomed by the medical team for check-in and vital signs. They are then invited to settle into the room where the session will take place. Each room is set up with mats on the floor in a circular formation. A ceremonial process takes place, similar to the CoP structure, while also honoring local Indigenous and cultural traditions and participant preferences. Consent forms are collectively reviewed and signed. Ketamine is administered as either oral lozenges for the first session and intramuscularly for the second and third sessions. Participants are provided with eye coverings and synchronized headphones, ensuring that they are all listening to the same uniquely constructed, ketamine-specific playlist. When the effects of the ketamine begin to wane around the 90-min mark, the initial sharing begins. Upon completion and ceremonial close, vital signs are repeated, and participants are escorted to the person driving them home.

Post-KaT, to facilitate integration within the first 36 h where neuroplasticity is most pronounced (25), participants attend group sessions in addition to their weekly CoP. These additional post-KaT sessions promote further integration of insights and new ways of being in their day-to-day lives.

### Participant Feedback

“*Although nervous at first of the unknown, I have found the sessions absolutely mind shifting. The work that I have done in these sessions have made me able to process my trauma and move forward in a way that I never thought would be possible. It brought forward things that I didn't know were affecting me. The days after the KaT sessions, I processed the experience even further.”*

### Ketamine Administration

Participants are given a dose of between 1 and 1.5 mg/kg by intramuscular injection. The dosing range promotes a psychedelic mechanism and is determined in conversation with the participant, based on their goals, intentions, and comfort with non-ordinary states of consciousness. In session two and three, the dosing is adjusted to remain aligned with participant's goals and comfort level. Dissociation is a normal (and often helpful) side effect for many. Some also experienced nausea, vomiting, and/or hypertension, which was resolved through pharmaceutical and non-pharmaceutical approaches.

## Treatment Results

**Figure 1:**
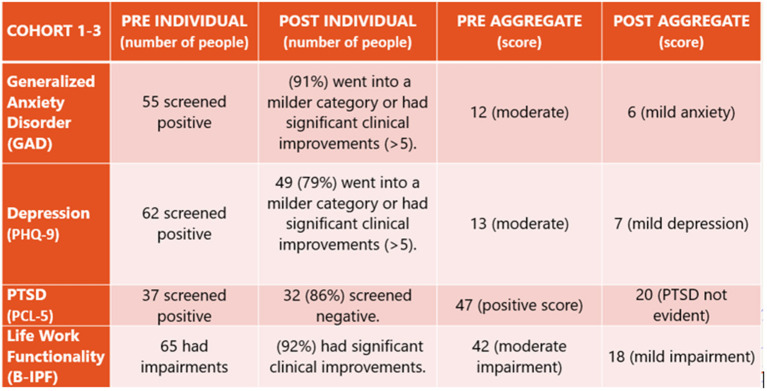
Cohort 1, 2, and 3 Combined Quantitative Results. The enclosed data includes the participants who screened positive for PTSD, depression, or generalized anxiety disorder upon entry into the RTI-KaT program. The pre-results were taken within 1 month of the program beginning and post-results were taken directly after the 12-week program was completed. If data was missing, it was not included.

**Figure 2:**
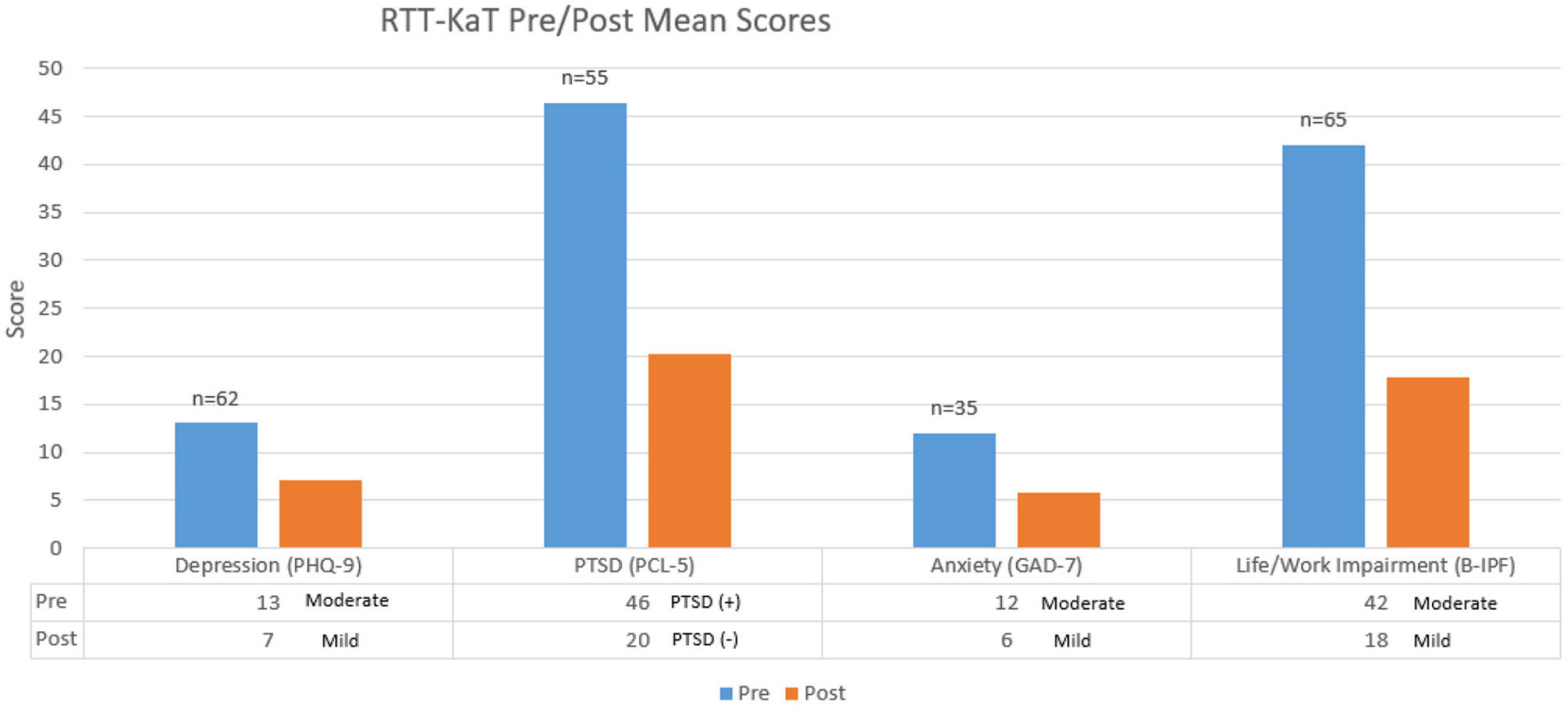
Cohort 1, 2, and 3 aggregate: mean scores. The “*n*” represents the number of participants who screened positive for PTSD, depression, or generalized anxiety disorder upon entry into the RTT-KaT program. The pre-results were taken within 1 month of the program beginning and post results were taken directly after the 12-week program was completed. If data was missing, it was not included.

Over 1 year, three cohorts totaling 94 participants completed the 12-week treatment program. With each cohort, quality improvement results were directly translated to the frontline of care and to ongoing program development efforts. As illustrated in Figures 1, 2, mean aggregate results of those who screened positive for GAD, Depression, PTSD, or Life/Work Impairments were clinically significant, demonstrated by a shift in score of five or greater after the 12-week treatment program.

- Of those who screened positive for generalized anxiety disorder symptoms (GAD), such as feeling on edge, not being able to stop worrying, trouble relaxing, unable to sit still, irritability, a sense of impending doom, 91% had reduced scores, dropping into a milder category or had significant clinical improvements.
- Of those who screened positive for symptoms of depression, such as feeling hopeless, little interest in doing things, sleeping too much or too little, loss of appetite and energy, trouble concentrating, feeling like a failure, and thoughts of being better off dead, 79% had reduced scores that dropped them into a milder category or had significant clinical improvements.
- Of those who screened positive for PTSD, such as re-experiencing past, emotional numbness, avoidance of people and activities that remind them of past trauma, difficulty sleeping, jumpy, easily irritated and angered, 86% left the program screening negative for PTSD.
- Of those who screened positive for Life/Work Impairments in romantic relationships, family relationships, work, friendships and socializing, parenting, education, and self-care, 92% had significant clinical improvements.

### Participant Feedback

*Upon Completion in response to “What are you noticing about yourself and your relationships?”:*

“*I have been led through some of my fears while experiencing a new found deep sense of love for life. I am able to connect with my partner from a more intimate authentic place. I am also noticing a greater depth of connection with those I work with and for.”*

“*I've stopped using Edibles [Cannabis]. I've felt more empowered, like I have my voice. I'm more connected to myself. I am quicker to reach out. I find love easier in connection. I'm less anxious at work and have more ease as I enjoy my colleagues more and trust they enjoy me. I'm noticing more space within myself. I'm not engaging in the push-pull in my relationships as much.”*

### Limitations and Suggestions for Future Research

This cohort-based case study was completed within a registered quality improvement project with the local health authority, which was exempt from REB review. Running through a program of research with a control group (comparing the CoP alone with the CoP + KaP) will promote further understanding of impact factors.

## Conclusion

Access to ketamine assisted therapy is limited, the cost of treatment, and durability of results are common challenges. Using a group/CoP model reduces cost, enhances capacity and thereby access, accelerates recovery and may reduce relapse by providing ongoing integration support over the 12 weeks of the program and beyond. Based on the learnings and positive outcomes of this program, we anticipate that the demand for communities of practice as a treatment and supportive integration mechanism will become a gold standard in the delivery of psychedelic-assisted therapies. Finally, we would like to recognize the HCPs that courageously stepped forward to co-develop this program in the process of navigating their own healing journeys.

## Data Availability Statement

The original contributions presented in the study are included in the article/supplementary material, further inquiries can be directed to the corresponding author/s.

## Ethics Statement

The project was registered as a Quality Improvement Project with the local health authority, exempt from formal ethical review under TCPS 2 (2018), Article 2.5. The patients provided their written consent to participate in the program.

## Author Contributions

All authors provided substantial contributions to the conception or design of the work, or the acquisition, analysis or interpretation of data for the work, drafted the work or revising it critically for important intellectual content, provided approval for publication of the content, and agreed to be accountable for all aspects of the work in ensuring that questions related to the accuracy or integrity of any part of the work are appropriately investigated and resolved.

## Funding

The project was supported by Vancouver Island University, a Canadian Institutes of Health Research Knowledge Synthesis grant, a British Columbia Ministry of Health COVID-19 Research grant, the Regional Initiatives Fund, in-kind support from Island Health, the British Columbia Nurses Union, First Nations Health Authority, and philanthropic funds.

## Conflict of Interest

The authors declare that the research was conducted in the absence of any commercial or financial relationships that could be construed as a potential conflict of interest.

## Publisher's Note

All claims expressed in this article are solely those of the authors and do not necessarily represent those of their affiliated organizations, or those of the publisher, the editors and the reviewers. Any product that may be evaluated in this article, or claim that may be made by its manufacturer, is not guaranteed or endorsed by the publisher.
