# Corrigendum: A cohort-based case report: The impact of ketamine-assisted therapy embedded in a community of practice framework for healthcare providers with PTSD and depression

**DOI:** 10.3389/fpsyt.2022.962882

**Published:** 2022-07-19

**Authors:** Shannon Dames, Pamela Kryskow, Crosbie Watler

**Affiliations:** ^1^Health and Human Services, Vancouver Island University, Nanaimo, BC, Canada; ^2^Private Practice, Duncan, BC, Canada

**Keywords:** ketamine-assisted psychotherapy, healthcare providers (HCP), resilience, psychedelic therapy, group therapy, community of practice, post-traumatic stress disorder (PTSD), depression

In the published article, there was an error in Figure 2 as published. The PTSD labels in the pre- and post- rows of the figure were incorrectly positioned underneath the Anxiety score, and vice versa. The corrected version of Figure 2 and its caption is published below.

**Figure 2:**
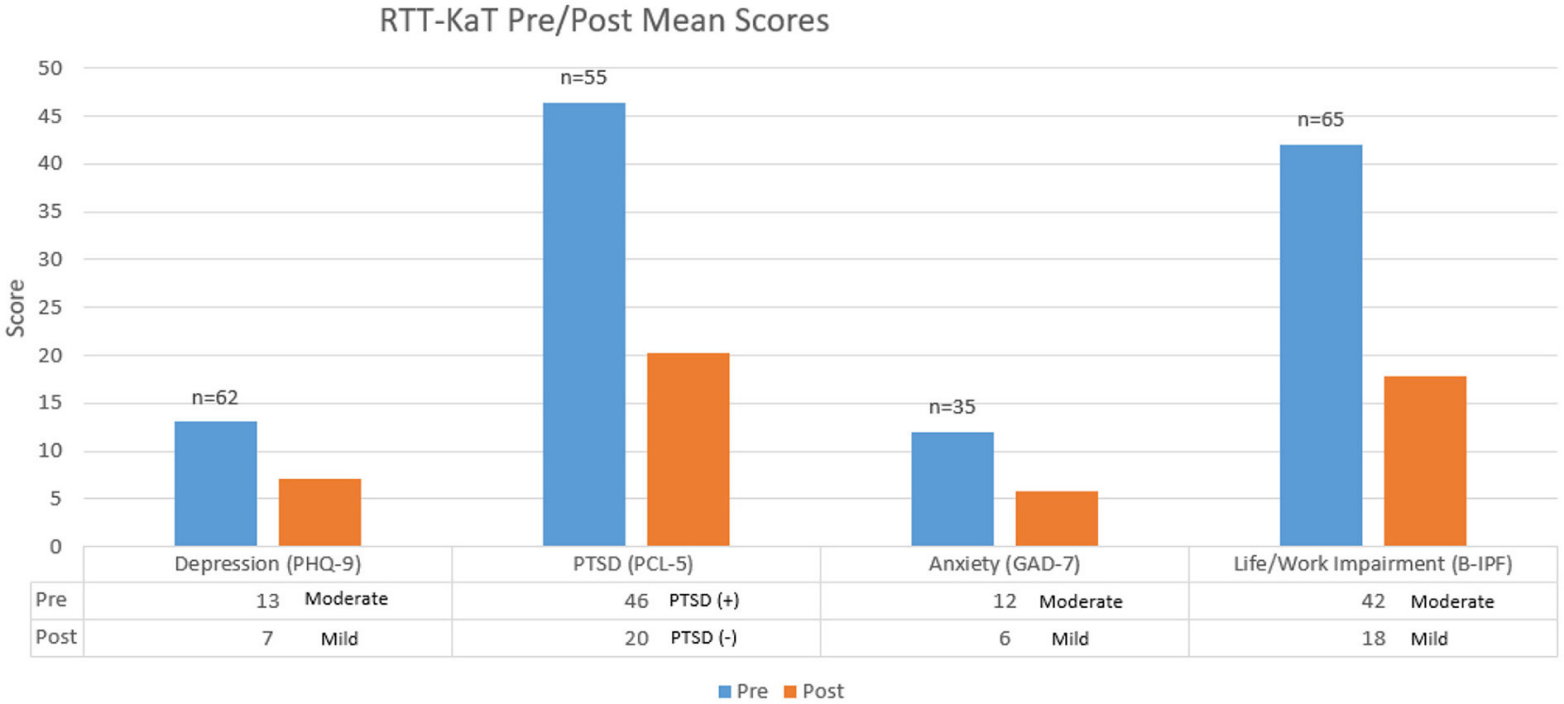
Cohort 1, 2, and 3 aggregate: mean scores. The “*n*” represents the number of participants who screened positive for PTSD, depression, or generalized anxiety disorder upon entry into the RTT-KaT program. The pre-results were taken within 1 month of the program beginning and post results were taken directly after the 12-week program was completed. If data was missing, it was not included.

The authors apologize for this error and state that this does not change the scientific conclusions of the article in any way. The original article has been updated.

## Publisher's note

All claims expressed in this article are solely those of the authors and do not necessarily represent those of their affiliated organizations, or those of the publisher, the editors and the reviewers. Any product that may be evaluated in this article, or claim that may be made by its manufacturer, is not guaranteed or endorsed by the publisher.

